# Multifractal Analysis of Choroidal SDOCT Images in the Detection of Retinitis Pigmentosa

**DOI:** 10.3390/tomography10040037

**Published:** 2024-03-29

**Authors:** Francesca Minicucci, Fotios D. Oikonomou, Angela A. De Sanctis

**Affiliations:** 1 Department of Life, Health and Environmental Sciences, University of L’Aquila, 67100 L’Aquila, Italy; francesca.minicucci@gmail.com; 2 Department of Physics, University of Patras, 26504 Rio, Greece; pheconom@upatras.gr; 3 Department of Business Economics, University “G. D’Annunzio” of Chieti-Pescara, 65127 Pescara, Italy

**Keywords:** retinitis pigmentosa, choroid, spectral domain optical coherence tomography, multifractal analysis, generalized Renyi point-centered dimensions

## Abstract

The aim of this paper is to investigate whether a multifractal analysis can be applied to study choroidal blood vessels and help ophthalmologists in the early diagnosis of retinitis pigmentosa (RP). In a case study, we used spectral domain optical coherence tomography (SDOCT), which is a noninvasive and highly sensitive imaging technique of the retina and choroid. The image of a choroidal branching pattern can be regarded as a multifractal. Therefore, we calculated the generalized Renyi point-centered dimensions, which are considered a measure of the inhomogeneity of data, to prove that it increases in patients with RP as compared to those in the control group.

## 1. Introduction

Retinitis pigmentosa is a rare hereditary degenerative pathology of the chorio-retina, characterized by the presence of pigment in the retina. Its clinical presentation can be extremely variable from patient to patient, though it maintains some common characteristics, Verbakel et al. and Marigo [1,2]. It is caused by a mutation of a gene involved in the molecular cycle of vision, performed by retinal photoreceptors, Ferrari et al. [3].

The clinical diagnosis of retinitis pigmentosa is based on

- Night blindness (nyctalopia), that is, difficulties with night vision;
- Reduced visual acuity;
- A typical hyperpigmentation of the retina in a “bone spicule” pattern in the mid-periphery, visible by fundoscopy;
- Attenuation of retinal arteries;
- Dysfunction of photoreceptors, distinguishable by electroretinographic abnormalities;
- A peripheral ring scotoma and narrowing of the visual field (detectable by visual field testing).

Histologically, RP is identified by rod photoreceptor death followed by cone photoreceptor death at the more advanced stages of the disease, resulting in a thinning of the outer retinal layers and retinal vessels, waxy pallor of the optic nerve, and pigmentary changes in the retina.

Several genes, if mutated, can lead to RP, but a mutation in any of those genes is enough to cause the illness, and this also explains why this pathology is so heterogeneous. RP is usually classified into three subtypes according to the inheritance model: autosomal dominant, autosomal recessive, and X-linked.

The clinical variants, syndromic and non- syndromic, have a variable prevalence in the different populations studied. The global prevalence is 1 case for each 3000–5000 inhabitants (about 1.5 million cases in the world).

Early diagnosis of RP represents the actual and only defense against this illness. Because neither complete healing nor sight recovery is possible, the only possibility for a cure is to slow down the illness progression through the daily assumption of vitamin A, omega-3, and lutein. The most recent and advanced kind of treatment is gene therapy, achieved using stem cells.

We recall that the eye is composed of three overlapping layers from the innermost to the outermost: retina, uvea, and sclera. The retina is the innermost layer of the eyeball and is constituted by neurons. It is sensitive to light stimuli, and this is where the visual process starts. The choroid is part of the uvea; its nature is essentially vascular, and its task is to supply the retina, on the inner side, and the sclera, on the outer side, Campbell et al. [4].

The retina is of fundamental importance to ophthalmologists because it is where the visual process begins; therefore, retinal diseases are the main cause of blindness in the world.

The retinal vascular system appears as a sophisticated network of blood vessels. The development of such a network tends to seek configurations that minimize operational energy expenditure to deliver nutrients and carry away waste. Often, vascular diseases can manifest as abnormalities in this network, and thus retinal vascularization offers a way to acquire insight into the presence (or absence) of disease.

**Figure 1:**
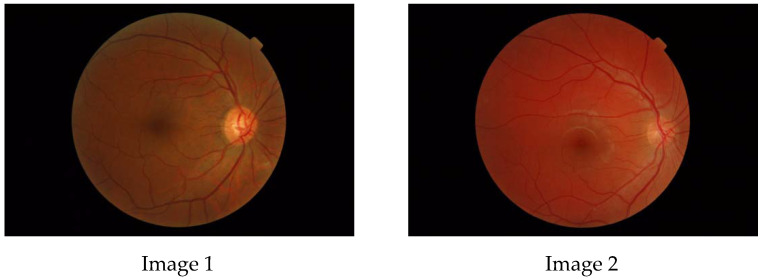
Images of healthy eyes.

From the geometrical point of view, the typical branching shape of retinal blood vessels is a fractal, Family et al. [5], which means an object that reproduces the same pattern on different levels of scale. As an example, the inexperienced viewer can consider the following images (Figure 1) of a healthy eye fundus of the posterior pole, which are found in the Messidor Database (kindly provided by the Messidor program partners (see https://www.adcis.net/en/third-party/messidor/. Accessed on 3 April 2021)) Decencière et al. [6].

The fractal dimension (FD), as in Lakshminarayanan et al. [7], provides a measure of the fractal’s global complexity. It is well known that for these kinds of objects, the dimension is not an integer number. Because the retinal blood vessels grow through the diffusion of angiogenic factors in the retinal plane, the fractal dimension lies between 1 and 2: that means its branching pattern fills space more than a line, but less than a plane. In healthy human subjects, the retinal FD has been proven to be around 1.7, but can be altered by the rarefaction or proliferation of blood vessels in the disease scenario. In Yu et al. [8], the authors summarize the current scientific literature on the association between FD and retinal disease.

The main imaging techniques to study the chorio-retina and that are therefore used in RP diagnosis, see Mitamura et al. [9], are

- fundus image photography;
- optical coherence tomography (OCT).

The first technique is the traditional way to identify abnormalities in the eye fundus.

Optical coherence tomography (OCT) is a new noninvasive diagnostic imaging modality enabling in vivo three-dimensional (3D) visualization of tissue microstructure, Fujimoto et al. [10]. Novel high-speed detection techniques and light source technology have revolutionized imaging performance and clinical feasibility of OCT, paving the way for cellular resolution retinal imaging and wide-field 3D choroidal visualization for routine clinical diagnosis.

Furthermore, extensions of OCT have been developed that enable noninvasive depth-resolved functional imaging of the retina, providing spectroscopic, blood flow, and physiologic tissue information. These extensions should not only improve the image contrast but also enable the differentiation of retinal pathologies through localized functional states.

Among these, optical coherence tomography angiography (OCTA) allows for the indirect visualization of the chorio-retinal vessels through the normal movement of blood in the capillaries, Jauregui et al. [11]. Compared with fluorescein angiography (FA), the advantages of OCTA are that it is noninvasive, acquires volumetric scans that can be segmented to specific depths, uses motion contrast instead of intravenous dye, can be obtained within seconds, provides accurate size and localization information, and visualizes both the retinal and choroidal vasculature. We can distinguish

○ OCTA small field, which is limited to the posterior pole (macula and optic discs);
○ OCTA wide field, which allows for the analysis of a larger retinal area.

Therefore, OCTA wide field is an imaging modality useful for the evaluation of RP disease, which mainly affects the periphery of the eyes.

In Minicucci et al. [12], we considered the images from OCTA wide field of chorio-retinal vessels at three different levels: superficial capillary plexus, deep capillary plexus, and choriocapillaris plexus of healthy subjects and retinitis pigmentosa (RP) patients. The images were referred to an experiment of 12 patients with a previous diagnosis of either mid- or late-stage RP and a control group of 20 healthy age-matched subjects, at the University “G. d’Annunzio” of Chieti-Pescara, Italy.

**Figure 2:**
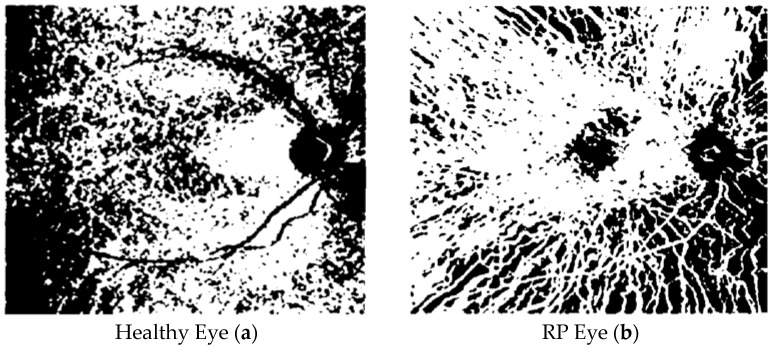
OCTA binary images in the choriocapillaris plexus of a healthy eye (**a**) and of an RP eye (**b**).

We noticed that the main feature of the OCTA images is that they present different levels of gray, which go from black to white; therefore, by setting a threshold parameter for the gray level, we have a binary image with its own FD. In Figure 2, as an example, we show the two OCTA binary images in the choriocapillaris plexus of a healthy eye and an eye with RP, corresponding to a particular threshold parameter for the gray. In Minicucci et al. [12], we have shown that, even when considering the possible variations indicated by the error bars, for the best possible binary images, the FD of the images of healthy eyes is more than that of the images of patients with RP. For very small or very large values of the threshold parameter, we have a very distorted image, and the two curves seem almost coincident.

In the present paper, we will deal with another imaging technique: the spectral domain optical coherence tomography (SDOCT), which provides high-resolution, optical cross-sectional, and en-face analysis of the retina and choroid with depth-resolved segmentation, Spaide et al. [13]. SDOCT guarantees excellent focus with high-resolution visualization of the retina. There is a progressive decrease in signal toward the choroidal structures resulting in a decrease in the sensitivity and the resolution of images moving away from the zero-delay point (which corresponds to the inner retinal edge). There is a limitation of the dynamic range offered by the analog to digital conversion before the Fourier transformation, and the wavelength-dependent light scattering induces a reduction in the signal noise contribution in the information coming from the lower retinal layers. An accurate study of the choroid is possible by performing SDOCT with enhanced depth imaging (EDI) technology, where the scan is closer to the eye of the patient to obtain an inverted image showing the deeper retinal layers and the choroid closer to the zero-delay point. This permits an enhanced and high-resolution image of the choroid up to the inner portions of the sclera, Margolis et al. and Chhablani et al. [14,15]. Therefore, information on retinal architecture can be integrated with that on choroidal vasculature to better understand the pathogenesis of RP, Zhang et al. [16].

In addition to the previous OCT techniques, it is worth mentioning two emerging optical modalities for the detection of RP disease, which has already been proven in animal models. The modalities are directional optical coherence tomography (dOCT) and multimodal retinal imaging, reported by Meleppat et al. [17,18,19].

Choroid assessment in patients with RP has been the subject of study for several years, Finzi et al. and Strobbe et al. [20,21]. The role of the choroid in the pathogenetic mechanism of retinal damage in RP is not clear, but it is now accepted that choroidal alterations participate as the primum movens of damage. Impairment of the choroidal circulation leads to a reduction in the intraretinal vascular flow and, invariably, to retinal photoreceptor damage. The reduction in choroidal vascularization and the reduction in retinal flow are probably associated with high values of plasma endothelin-1 (ET) in patients with RP.

**Figure 3:**
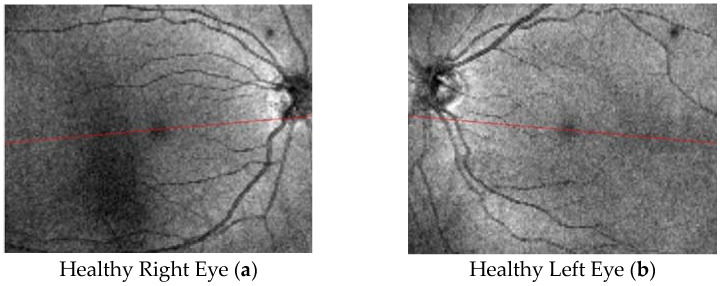
SDOCT choroidal images referring to healthy eyes (right eye (**a**) and left eye (**b**)).

In the present study, we use spectral domain optical coherence tomography (SDOCT) to obtain choroidal images. The following SDOCT choroidal images (Figure 3) refer to healthy eyes (right eye (a) and left eye (b)) and were kindly provided by the group of Prof. S. Abdolrahimzadeh in “Sant’Andrea” Hospital of Rome (IT).

In Section 2 of the paper, we present the mathematical background, recall briefly fractals and fractal dimensions, multifractals, and generalized Renyi point-centered dimensions. Readers not interested in definitions and mathematical details may omit this section and go directly to Section 3, where we present the algorithms we used to calculate such dimensions.

In Section 4, we present an experiment with two healthy subjects (control group) and three RP patients, which was performed by the group of Prof. S. Abdolrahimzadeh at “Sant’Andrea” Hospital of Rome (IT). We analyzed the choroid vessels using the SDOCT technique. We first deduced the FD of the choroidal imaging for each pair of eyes, finding that it decreases in RP patients and not in healthy subjects. In addition, due to the choroidal vascular network being a more proper multifractal pattern, we calculated the generalized Renyi point-centered dimensions, finding that they are lower for the control group and increase in the patients with RP. At the end, there will be a discussion of the results obtained, and conclusions will be drawn.

## 2. Mathematical Background

This section may be omitted on a first reading.

### 2.1. Fractal Dimension

In this paragraph, we briefly revisit the concept of fractal dimension, and for a more in-depth exploration of fractals and fractal dimension, please refer to Mandelbrot et al., Falconer, Barnsley and Peitgen et al. [22,23,24,25,26]. Let us consider an “object” existing in d dimensions. If d=1, this “object” may consist of a set of line segments; for d=2, it could be an assembly of parts from a plane; for d=3, it might represent a section of three-dimensional space, and so on. It is widely acknowledged that we can attribute a “measure” M to this “object”. In the case of d=1, the “measure” corresponds to the length of the line segments; for d=2, it represents the area of the plane parts; for d=3, it signifies the volume of the three-dimensional part, and so forth.

To encompass this object, we can use “boxes” with a sufficiently small side length denoted as l. In the context of d=1, these “boxes” are small line segments; for d=2, they take the form of small squares; and for d=3, they manifest as small cubes. If N(l) represents the minimum number of boxes with a side length of l required to cover the object, it becomes evident that

$$M\approx N(l)l^{d}$$

given that $l^{d}$ serves as the length, area, or volume of each box. Solving the equation above in relation to d results in:

$$d\approx \frac{logM}{logl}+\frac{logN(l)}{log(l^{-1})}$$

where the logarithm can be in any base.

As l is a negligible value and M is a constant, the term logM/logl can be omitted. Hence,

$$d\approx \frac{logN(l)}{log{(l}^{-1})}$$

The calculated value of d from the formula above may not be an integer. To be more accurate, the number D

(1) $$D=\underset{l\to 0}{\lim}\frac{logN(l)}{log(l^{-1})}$$

can be any real number (less than the embedding dimension) and is termed the “Fractal Dimension” of the object mentioned above, see Mandelbrot et al., Falconer, Barnsley, and Peitgen et al. [23,24,25,26].

### 2.2. Multifractals

Let us examine a probability space denoted as $\left(X,B\left(X\right),\mu \right)$. Assume $X\subset R^{d}$, and B(X) represents the Borel subsets of X. Our focus is on situations where the probability measure μ on B(X) displays a high degree of irregularity, and the distribution is non-differentiable, with singularities of possibly many different orders. Such a measure is termed multifractal, Harte [27].

Consider a lattice that encompasses the support of μ using d-dimensional boxes of side l. The box that contains x is denoted by $B_{l}(x)$, where $x\in X_{l}$ and $X_{l}=\left\{x:\mu \left[B_{l}\left(x\right)\right]>0\right\}$. We evaluated successive lattice coverings for l⟶0.

Let

(2) $$K_{l}\left(y,\varepsilon \right)=\left\{x:y-\varepsilon <\frac{\log\mu [B_{l}\left(x\right)]}{\log l}\leq y+\varepsilon \right\}$$

The multifractal spectrum denoted by $\tilde{f}(y)$ is defined to be

$$\tilde{f}\left(y\right)=\underset{\varepsilon \to 0}{\lim}\underset{l\to 0}{\lim}\frac{\log\#K_{l}\left(y,\varepsilon \right)}{\log\left(l^{-1}\right)}$$

for y>0. It is clear that this relation corresponds to (1) above. We will consider the Legendre transformation of $\tilde{f}(y)$

$$\tilde{\theta }\left(q\right)=\underset{y}{\underbrace{inf}}\left\{qy-\tilde{f}(y)\right\}$$

or equivalently

$$\tilde{\theta }\left(q\right)=qy-\tilde{f}(y)\;\mathrm{and}\;\frac{\partial \tilde{\theta }}{\partial q}=y,\;\frac{\partial \tilde{f}}{\partial y}=q$$

From the last one, it is clear that if $\tilde{\theta }$ and $\tilde{f}$ depend on some parameter *λ* connected to subjects who may or may not be healthy, we have

$$\frac{\partial^{2}\tilde{\theta }}{\partial \lambda^{2}}=-\frac{\partial^{2}\tilde{f}}{\partial \lambda^{2}}$$

Hence, for maximal values of $\tilde{f}(y)$, we will have minimal values of $\tilde{\theta }(q)$ and vice versa.

It can be proven that

$$\tilde{\theta }\left(q\right)=\underset{l\to 0}{\lim}\frac{\log\sum_{x\in X_{l}}\mu^{q}\left(B_{l}\left(x\right)\right)}{\log l}=\underset{l\to 0}{\lim}\frac{\log E(\mu^{q-1}\left(B_{l}\left(X\right)\right)}{\log l}$$

Instead of this, for the calculations, we consider the point-centered correlation exponents defined as

$$\theta \left(q\right)=\underset{l\to 0}{\lim}\frac{\log\left(\int_{X_{l}}\mu^{q-1}\left(S_{l}\left(x\right)\right)\mu (dx)\right)}{\log l}$$

where $S_{l}(x)$ is a sphere of radius l centered at x. Then the generalized Renyi point-centered dimensions are defined as

$$D_{q}=\left\{\begin{array}{c} \frac{\theta \left(q\right)}{q-1}\;\;if\;\;\;q\neq 1\\ \underset{l\to 0}{\lim}\frac{\int_{X_{l}}\log\left(\mu (S_{l}\left(x\right))\right)\mu (dx)}{\log l}\;\;if\;\;\;q=1 \end{array}\right.$$

**Theorem** **1.** *Consider* $X_{1},X_{2},\ldots ,X_{q}$ *as a sample of independent random variables selected from the probability distribution* μ*.* *Define* Y *as follows*

$$Y=max\left\{\left\|X_{1}-X_{q}\right\|,\left\|X_{2}-X_{q}\right\|,\ldots ,\left\|X_{q-1}-X_{q}\right\|\;\right\}$$

*where* $\left\|\cdot \right\|$ *is the maximum norm. Then, for* q=2,3,4,…

$$\int \mu^{q-1}\left(S_{y}\left(x\right)\right)\mu (dx)=Pr\left\{Y\leq y\right\}\equiv F_{Y}(y)$$

**Proof**  (Harte [27])**.**

Let **1**(A) be one if A is true and zero otherwise; then, it is obvious that

$$\mu \left[S_{y}\left(x\right)\right]=\int 1\left(\left\|x_{1}-x\right\|\leq y\right)\mu \left(dx_{1}\right)=Pr\left\{\left\|X_{1}-x\right\|\leq y\right\}$$

Further,

$$\mu^{q-1}\left[S_{y}\left(x\right)\right]={\left[\int 1\left(\left\|x_{1}-x\right\|\leq y\right)\mu \left(dx_{1}\right)\right]}^{q-1}\;=\int \ldots \int 1\left(\left\|x_{1}-x\right\|\leq y\right)\cdots 1\left(\left\|x_{q-1}-x\right\|\leq y\right)\mu \left(dx_{1}\right)\cdots \mu \left(dx_{q-1}\right)\;=\int \ldots \int 1(max\{\left\|x_{1}-x\right\|,\ldots ,\left\|x_{q-1}-x\right\|\}\leq y)\mu \left(dx_{1}\right)\cdots \mu \left(dx_{q-1}\right)$$

so,

$$\int \mu^{q-1}\left[S_{y}\left(x\right)\right]\mu (dx)\;=\int \int \ldots \int 1(max\{\left\|x_{1}-x_{q}\right\|,\ldots ,\left\|x_{q-1}-x_{q}\right\|\}\leq y)\mu \left(dx_{1}\right)\cdots \mu \left(dx_{q-1}\right)\mu \left(dx_{q}\right)\;=Pr\left\{Y\leq y\right\}$$

□

Especially for *q* = 2, we have

$$Pr\left\{\left\|X_{1}-X_{2}\right\|\leq l\right\}=\int \mu \left(S_{l}\left(x\right)\right)\mu (dx)$$

which is more or less expected.

From the above relations, we have for q=2,3,4,…,

$$\theta \left(q\right)=\underset{y⟶0}{\lim}\frac{\log F_{Y}(y)}{\log y}$$

**Theorem** **2.** *The correlation dimension exists if the correlation integral* $F_{Y}(y)$ *can be decomposed in the following form:*

$$F_{Y}\left(y\right)=\Phi (y)y^{\zeta_{q}}$$

*where* $\zeta_{q}$ *is a positive constant and* Φ(y) *is a positive function such that*

$$\underset{y⟶0}{\lim}\frac{\log\Phi (y)}{\log y}=0$$

*Further, given this decomposition,* $\theta \left(q\right)=\zeta_{q}$.

The analysis follows mainly Harte [27].

## 3. The Algorithms

### 3.1. Computing the Fractal Dimension

To compute the fractal dimension of the images, we analyzed them first using mathematica, in order to extract the tree-shaped structure of the blood vessels. We checked each pixel of the image, and if it belonged to a blood vessel (if it was black), we passed to the next pixel(s) of the particular blood vessel by examining the neighboring pixels, reconstructing in that way the blood vessel. Then, we proceeded with fractal dimensional box-counting analysis as in Falconer [24], which is performed using Fractalyse (ThéMA, Besançon Cedex, France). Fractalyse uses linear regression, so, possible noise in the data (images) minimally affects the results. In addition, as shown by Reiss et al. [28], noise may only increase the FD of an image. Because we deal with comparisons of FDs of images, our conclusions are not altered because of possible noise.

### 3.2. Computing the Generalized Renyi Point-Centered Dimensions

#### 3.2.1. The Case When q=2

Grassberger and Procaccia investigated the scenario with q=2. Given a finite sequence of vector random variables $X_{1},X_{2},\ldots ,X_{N}$ in $R^{d}$, their approach involves computing all conceivable interpoint distances and using these to construct an empirical distribution function as an estimate of $F_{Y}(y)$. That is, for q=2,

$${\hat{F}}_{Y}^{GP}\left(y,N\right)=\frac{2}{N(N-1)}\sum_{i=1}^{N-1}\sum_{j=i+1}^{N}1\left(\left\|X_{i}-X_{j}\right\|\leq y\right)$$

where **1**(A) is one if A is true and zero otherwise. The procedure then involves plotting logy by sample values of $\log{\hat{F}}_{Y}^{GP}\left(y,N\right)$ and using the slope of the line in some suitable region as an estimate of the correlation dimension $D_{2}=\theta (2)$.

Our final step deviated slightly. We found the ${\hat{F}}_{Y}^{GP}\left(y,N\right)$ as above for the discrete values of y and fitted to the data the expression $ay^{b}$ using mathematica. Then, according to Theorem 2 above $\theta \left(q\right)=b$.

#### 3.2.2. The Case When q=2,3,…

Given a finite sequence of vector random variables $X_{1},X_{2},\ldots ,X_{N}$ in $R^{d}$, we defined the random function ${\hat{F}}_{Y}^{GP}\left(y,N\right)$ as

$${\hat{F}}_{Y}^{GP}\left(y,N\right)=\frac{1}{m}\sum_{k=1}^{m}1\left(Y_{k}\leq y\right)$$

where $Y_{1},Y_{2},\ldots ,Y_{m}$ are all possible permutations of the *q*th order difference given a sample of *N* points, the *k*th being

$$Y_{k}=\max\left\{\left\|X_{k_{1}}-X_{k_{q}}\right\|,\left\|X_{k_{2}}-X_{k_{q}}\right\|,\ldots ,\left\|X_{k_{q-1}}-X_{k_{q}}\right\|\right\}$$

In cases where *q* > 2, one samples $X_{k_{q}}$ from $X_{1},X_{2},\ldots ,X_{N}$ without replacement. Subsequently, $X_{k_{i}}$ for i=1,2,…,q−1 is sampled with replacement. In this second phase, there are $N^{q-1}$ possibilities, and in the first phase, there are *N* possibilities. Therefore, $m=N{\left(N-1\right)}^{q-1}$. The function ${\hat{F}}_{Y}^{GP}\left(y,N\right)$ is an estimator of $F_{Y}(y)$.

We implemented the above algorithm as follows. We know that every image is a collection of pixels, actually a 2-dimensional array of pixels. Every pixel has a luminosity equal to some value. So, a picture corresponds to a matrix with elements of pixel’s luminosities. We normalized these values and interpolated between them, so we had a 2-dimensional probability density function, i.e., a probability distribution μ. Then, we gave values to $X_{1},X_{2},\ldots ,X_{N}$ for N=100 according to the distribution μ and computed as above the ${\hat{F}}_{Y}^{GP}(y,N)$ for 10 values of y which is an estimation of $F_{Y}(y)$.

Finally, we fitted to the points of ${\hat{F}}_{Y}^{GP}(y,N)$ the expression $ay^{b}$ for a, b constants using mathematica. Then, according to the above theorem, 2 $\theta \left(q\right)=b$. The fitting procedure minimized the impact of possible noise on the results.

## 4. Results

In the present paper, we consider two healthy subjects (control group), who will be called “Subject 1” and “Subject 2”. We also consider three patients:

- A patient with homozygous PDE6b mutation corresponding to the images of Figure 1a,b of Abdolrahimzadeh et al. [29] and is called “Patient 1”;
- A myopic patient with rhodopsin rhoAsp190Asn mutation corresponding to Figure 2a,b of Abdolrahimzadeh et al. [29] and is called “Patient 2”;
- A patient with Usher’s disease corresponding to Figure 3a,b of Abdolrahimzadeh et al. [29] and is called “Patient 3”. Usher’s syndrome is a genetic disease characterized by partial or total loss of hearing and vision, due to anomalies of the inner ear and retina, respectively. It is considered the most frequent cause of blindness associated with early-onset deafness and has an estimated prevalence of around one case in 30,000 people. Three types can be distinguished based on the symptoms and age of onset. Type 1 presents with severe deafness from birth and a progressive loss of vision starting from childhood, as well as disorders of balance and orientation in space. Children with this form in fact begin to walk later and may have difficulty riding a bicycle or playing some sports. There are at least 10 genes associated with Usher’s syndrome, all involved in the production of proteins aimed at the processes of vision, hearing, and balance. In all cases, the syndrome is transmitted in an autosomal recessive manner.

**Figure 4:**
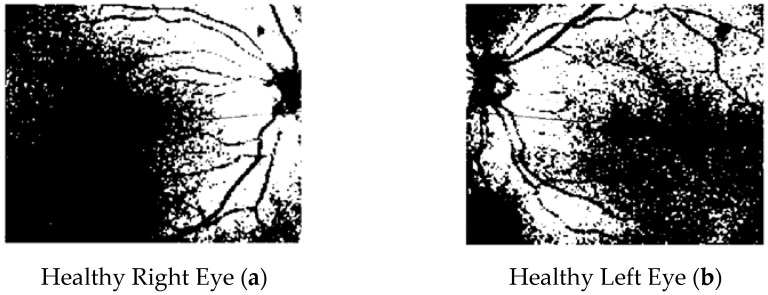
SDOCT binary choroidal images referring to healthy eyes (right eye (**a**) and left eye (**b**)).

In the following figure (Figure 4), there are the binarized SDOCT images of a healthy subject.

**Figure 5:**
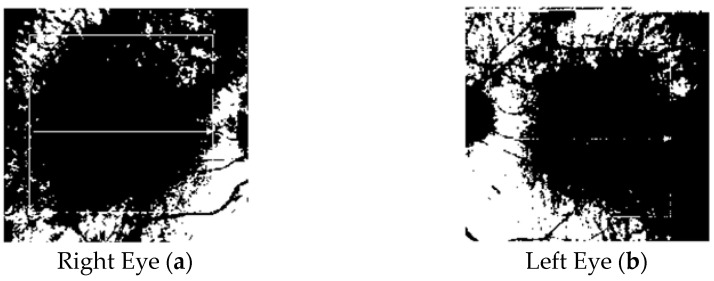
Near-infrared reflectance and spectral domain optical coherence tomography scans in a patient with Usher’s disease (right eye (**a**) and left eye (**b**)).

And in Figure 5, the images are of a patient with Usher’s syndrome.

### 4.1. Fractal Dimension

For the previous two healthy subjects and three patients, we first deduced the FD of the choroid vessels.

We obtained the results shown in the Table 1 and Table 2 below, where r^2^ is a measure of the quality of the linear regression analysis in our model (r^2^ = 1 means best fitting). Fractalyse uses the *p*-value approach to hypothesis testing. Because *p*-values are very small, there is strong evidence that logN(l) and $log(l^{-1})$ are linearly related.

**Figure 6:**
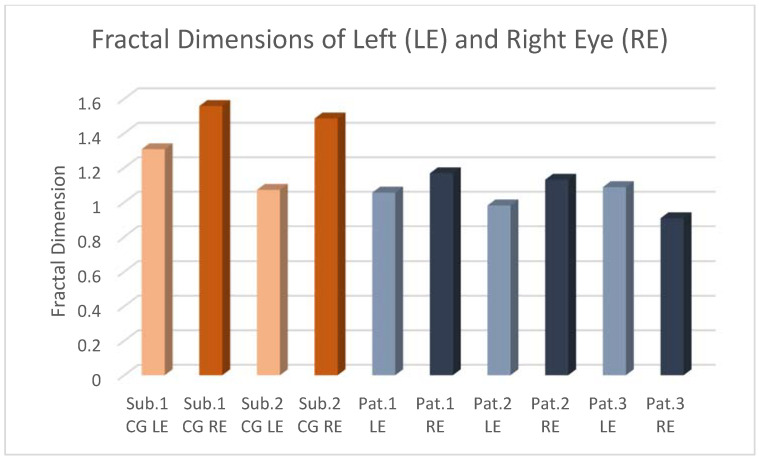
Fractal dimensions of healthy subjects and patients of Table 1 and Table 2.

The FDs of Table 1 and Table 2 are shown in Figure 6.

We can observe that, even if the literature refers to RP as a bilateral pathology, for these patients, it seems that the right eye is mostly affected. In addition, among the three pathologies, Usher’s disease seems to be the most serious.

### 4.2. Generalized Renyi Point-Centered Dimensions

A multifractal set can be seen as the union of several mono-fractal subsets, among them freely interconnected, each having its own dimension fractal.

Such a union can be described entirely by an infinite number of generalized dimensions D, also called Renyi’s, i.e., across the spectrum f, known as the multifractal spectrum.

Another important index, used in the literature for multifractal analysis, is the generalized Renyi point-centered dimensions, which represent a measure of inhomogeneity of data, Abdolrahimzadeh et al. [29].

The chorio-retinal vascular network could be more properly considered a multifractal pattern because its different regions have different fractal properties characterized by an infinite number of generalized dimensions, rather than a single fractal dimension.

**Figure 7:**
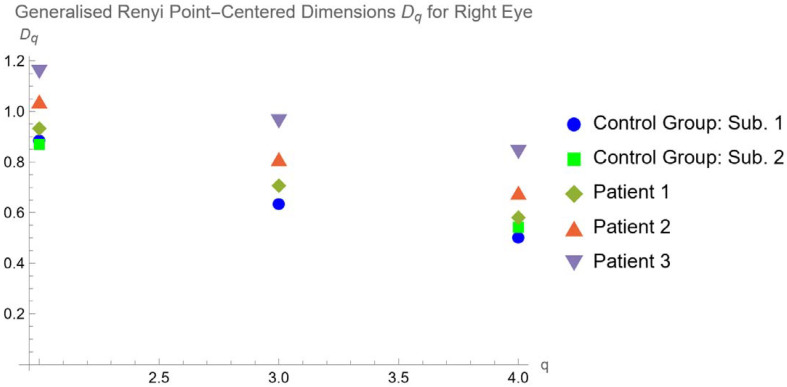
Generalized Renyi Point-Centered dimensions for right eye of healthy subjects and patients.

**Figure 8:**
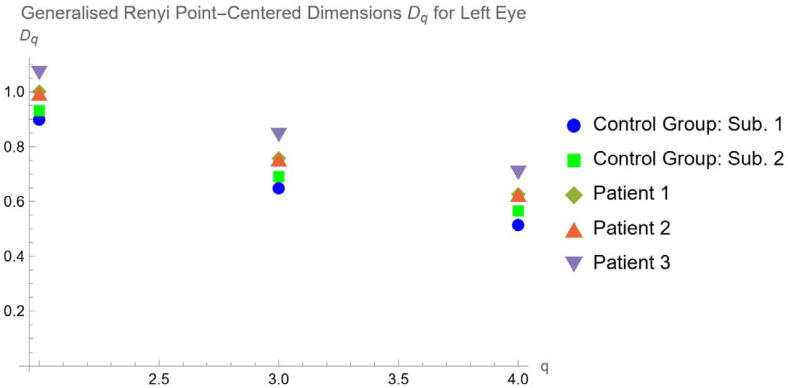
Generalized Renyi Point-Centered dimensions for left eye of healthy subjects and patients.

Therefore, we calculated the generalized Renyi point-centered dimensions for our case study. We obtained the results, which are summarized in the following graphs (Figure 7 and Figure 8):

## 5. Discussion

In the present experiment, we analyzed a case study of two healthy subjects and three RP patients. We studied the choroid’s vessels using the SDOCT technique. We first deduced the FD of the choroidal imaging for each pair of eyes, finding that it decreases in RP patients as opposed to healthy subjects. This means that, in the genetic pathology of retinitis pigmentosa, the tree of choroidal vascularization has fewer branching connections; therefore, the geometry of the choroid becomes more simplified. Consequently, complete and efficient vision is not possible in patients from a young age, and the visual ability degenerates further with aging.

In addition, due to the choroidal vascular network being a more proper multifractal pattern, we calculated the generalized Renyi point-centered dimensions, finding that they are lower for the control group and increase in the patients with RP, mostly in Usher’s cases. This is compatible with the mathematical dependence between FD and generalized Renyi point-centered dimensions.

## 6. Conclusions

New imaging techniques together with computer science and multifractal analysis can be very useful in RP disease for early diagnosis, which represents the only defense against this rare genetic illness. According to the clinical observation that choroid alterations of vascular circulation lead to photoreceptor damage, we focused on choroidal images, using the new technique of spectral domain optical coherence tomography (SDOCT).

Due to the multifractal structure of the choroid blood vessels, we used the generalized Renyi point-centered dimensions as a measure of the inhomogeneity of data. We showed, in a study case, that the generalized Renyi point-centered dimensions of the choroidal images increase in patients with RP as compared to healthy subjects. It could be due to the increase in inhomogeneity in the images. Although the small number of samples is not statistically significant, this result enables us to propose the generalized Renyi point-centered dimensions as an index to warn of the presence of RP pathology.

As a future work, we aim to perform a study on a number of patients to allow for a quantitative comparative estimation and a complete statistical analysis. It would be important to set a percentage of comparison with other non-genetical chorio-retinal pathologies such as diabetic retinopathy or glaucoma. In general, we can suppose from our research that the generalized Renyi point-centered dimensions of the choroidal images in RP, in mature age, are higher as compared with those of a non-genetic illness.

Before moving on to clinical applications, it is necessary that a standardized protocol for image acquisition/processing be established first to facilitate inter-study comparison.

## Figures and Tables

**Table 1:** Fractal dimensions for right eye of healthy subjects and patients.

Image	FD	$r^{2}$	Confidence (95%)	*p*-Value
Subject 1 (CG)	1.557	0.999	1.510–1.604	2.308 × 10^−10^
Subject 2 (CG)	1.485	1.000	1.456–1.514	1.838 × 10^−11^
Patient 1	1.167	0.997	1.096–1.237	1.350 × 10^−7^
Patient 2	1.130	0.999	1.083–1.177	2.092 × 10^−8^
Patient 3	0.907	0.992	0.815–0.998	1.758 × 10^−6^

**Table 2:** Fractal dimensions for left eye of healthy subjects and patients.

Image	FD	$r^{2}$	Confidence (95%)	*p*-Value
Subject 1 (CG)	1.307	0.989	1.172–1.443	3.803 × 10^−7^
Subject 2 (CG)	1.071	0.999	1.034–1.108	5.337 × 10^−10^
Patient 1	1.055	0.996	0.981–1.128	2.733 × 10^−7^
Patient 2	0.981	0.999	0.944–1.018	1.328 × 10^−8^
Patient 3	1.087	0.990	0.964–1.211	3.172 × 10^−6^

## Data Availability

The (SDOCT) images were kindly provided by the group of S. Abdolrahimzadeh in “Sant’Andrea” Hospital of Rome (IT).
